# Quantitative assessment of DNA replication to monitor microgametogenesis in *Plasmodium berghei*

**DOI:** 10.1016/j.molbiopara.2009.08.004

**Published:** 2009-12

**Authors:** Andreas C. Raabe, Oliver Billker, Henri J. Vial, Kai Wengelnik

**Affiliations:** a CNRS UMR5235, Université Montpellier 2, Place Eugène Bataillon, 34095 Montpellier Cedex 5, France; b Imperial College London, Division of Cell and Molecular Biology, London SW7 2AZ, UK; c The Wellcome Trust Sanger Institute, Cambridge CB10 SA1, UK

**Keywords:** Malaria, Gametocyte, Transmission, Exflagellation

## Abstract

Targeting the crucial step of *Plasmodium* transition from vertebrate host to mosquito vector is a promising approach to eliminate malaria. Uptake by the mosquito activates gametocytes within seconds, and in the case of male (micro) gametocytes leads to rapid DNA replication and the release of eight flagellated gametes. We developed a sensitive assay to monitor *P. berghei* microgametocyte activation based on [^3^H]hypoxanthine incorporation into DNA. Optimal pH range and xanthurenic acid concentrations for gametocyte activation were established and the kinetics of DNA replication investigated. Significance of the method was confirmed using *P. berghei* mutants and the assay was applied to analyse the effect of protease inhibitors, which revealed differences regarding their inhibitory action. The developed method thus appears suitable for reproducible determination of microgametocyte activation, medium-throughput drug screenings and deeper investigation of early blocks in gametogenesis and will facilitate the analysis of compounds for transmission blocking activities.

## 1 Results

Only successful infection of the mosquito allows the malaria parasite to reproduce sexually and eventually infect new vertebrate hosts, thereby spreading the disease. Targeting this pivotal step in the *Plasmodium* life cycle with transmission blocking drugs or vaccines is still poorly explored even though it is a promising approach in the fight against malaria. The need to investigate the transmission blocking potential of antimalarial drugs is widely recognised as important for the elimination of malaria. The only cells capable of infecting the mosquito are the sexual blood stages, male (micro) and female (macro) gametocytes. Duration of their maturation is species dependent and can range from little more than the asexual cycle (26–30 h) for *P. berghei* to >10 days for *P. falciparum* [1–3]. Mature gametocytes are developmentally arrested while circulating in the vertebrate blood stream, but become activated in the midgut of the mosquito seconds after ingestion, forming male and female gametes. Macrogametocytes give rise to a single macrogamete while microgametocytes re-enter the cell cycle and replicate their genome to the octoploid level in only 10 min, making this a remarkably rapid process [4]. Three rounds of endomitosis and concomitant assembly of eight axonemes finally give rise to eight flagellated and motile microgametes (exflagellation). Fusion of micro- and macrogametes leads to formation of a diploid zygote that itself develops into the motile ookinete which escapes from the blood meal by penetrating the mosquito gut wall.

Gametocyte activation is triggered *in vitro* by simultaneous exposure to two stimuli: a drop in temperature of more than 5 °C [5] and xanthurenic acid (XA), the latter of which can be substituted by a pH shift from 7.5 to 8.0 [6–9]. The underlying molecular mechanisms are intensively investigated [10–12] given the critical role of gametocyte activation in transmission of malaria. The standard method to monitor successful gametogenesis is by counting exflagellation centres under the microscope. However, this method is labour-intensive, somewhat subjective, cannot be automated, and is thus not suited for medium-throughput applications. DNA replication during microgametogenesis has been investigated previously using DNA staining with various fluorescent dyes and subsequent analysis of individual cells by fluorescence microscopy or of cell populations by flow cytometry [4,10,11,13]. These methods gave fundamental insight into the kinetics and general parameters of microgametocyte DNA synthesis and allowed phenotypic analysis of mutants; however they are not easily adapted to higher throughput analyses. Here we present an assay adapted to the 96-well format to monitor activation of gametocytes based on incorporation of radioactive hypoxanthine into newly synthesised DNA of microgametes.

**Fig. 1:**
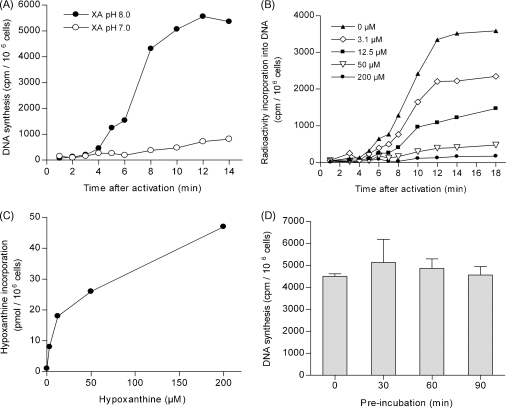
[^3^H]hypoxanthine incorporation into DNA during microgametogenesis. (A) Incorporation of [^3^H]hypoxanthine over time of gametogenesis. Gametocytes were purified as described previously [10] with minor modifications. Female NMRI mice (Charles River) were pre-treated with 0.1 ml phenylhydrazine (25 mg/ml in PBS) and infected two days later with 0.5–2 × 10^7^*P. berghei* ANKA clone 2.34 parasites from frozen blood stocks. On day 4 p.i. 20 mg/ml sulfadiazine in drinking water was applied to kill asexual stages. On day 6 p.i., mice were bled by cardiac puncture, the blood washed in gametocyte maintenance buffer (GMB: 4 mM sodium bicarbonate, 20 mM glucose, 137 mM NaCl, 4 mM KCl, 1 mM MgCl_2_, 1 mM CaCl_2_, 20 mM Hepes pH 7.24–7.29, 0.1% BSA) and purified on a 48% Nycodenz/GMB gradient (Nycodenz stock solution: 27.6% (w/v) Nycodenz in 3 mM KCl, 0.3 mM EDTA, 5 mM Tris–HCl pH 7.2). Gametocytes were resuspended in GMB and kept at 20 °C. As determined by Giemsa stained blood film, gametocytes were enriched to approximately 95% with contaminants being late stage trophozoites (≈4%), few red blood cells and occasionally some very few white blood cells. Gametocytes were activated at room temperature (22–26 °C) by transferring them to gametocyte activation medium (GAM; RPMI 1640 with 20 mM Hepes, 4 mM sodium bicarbonate, adjusted to pH 8.0 and supplemented with 100 μM XA) while the medium for controls was adjusted to pH 7.0. GAM was supplemented with 0.5 μM [^3^H]hypoxanthine (previously evaporated from 52 μM in water/ethanol 1:1, specific activity of 1 mCi/ml, GE Healthcare). 100 μl aliquots containing (3–15) × 10^6^ gametocytes were removed at the indicated time points and shock frozen in 96-well microtiter plates on liquid N_2_. Subsequently, macromolecules including DNA were recovered by filtering the lysates onto glass-fiber filter 96 plates (Perkin Elmer UniFilter 96 GF/C and Packard Filtermate Harvester). The membrane plate was washed 5 times by perfusion with H_2_O, bleached with 10% H_2_O_2_, dried at 50 °C for 30 min, loaded with 30 μl/well PerkinElmer Microscint 0, and the radioactivity determined in a TopCount NXT microplate scintillation counter (Packard Instruments). Values are expressed as cpm per 10^6^ cells and are averages of duplicates in one experiment representative of five. (B) Radioactive labelling of nucleic acids over time with fixed amount of radioactive [^3^H]hypoxanthine (0.5 μCi/well, 260 nM) supplemented with the indicated concentrations of cold hypoxanthine. (C) Total hypoxanthine incorporation during gametogenesis calculated from data shown in (B). (D) Effect of pre-incubation of gametocytes with 260 nM [^3^H]hypoxanthine before activation. Activation was performed at the indicate time and cells incubated for 20 min. Results are shown with S.E.M, *n* = 3. All graphs and analyses were generated using GraphPad Prism software.

The radioactive purine precursor [^3^H]hypoxanthine is readily metabolised into nucleotides by *Plasmodium* and routinely used to label DNA in asexual stages [14]. Given the considerable need for nucleotides during gametogenesis it seemed likely that gametocytes too would scavenge external hypoxanthine for incorporation into their DNA. Purified gametocytes were activated by transfer to gametocyte activation medium (GAM) in the presence of [^3^H]hypoxanthine and the incorporation of the radioactive label into macromolecules analysed. Strong radiolabel incorporation into nucleic acids was observed in gametocytes activated by a shift to pH 8.0 but not under non-activating conditions at pH 7.0 (Fig. 1A). At pH 8.0, label incorporation commenced after a short lag phase reaching an intermediate step after 6 min and continued to rise until a plateau was reached after 10 min. This pattern was repeatedly observed in independent experiments (data not shown). For one batch of purified gametocytes, we counted exflagellation events under the microscope and observed that at pH 8.0 exflagellation peaked at 14 min while no exflagellation was observed at pH 7.0 (data not shown). Thus, hypoxanthine incorporation correlated well with successful exflagellation.

Purines deriving from hypoxanthine can be incorporated into both DNA or RNA. Treatment of lysates with 10 μg RNaseA per 100 μl solution at 37 °C for 2 h completely removed RNA from non-radioactive control samples as detected by ethidium bromide staining of agarose gel separated nucleic acids. When lysates of [^3^H]hypoxanthine labelled parasites were digested with RNaseA before the macromolecules were filtered and analysed, we found no difference to untreated samples, showing that [^3^H]hypoxanthine was essentially incorporated into DNA (data not shown).

We also excluded the possibility that asexual stages, essentially late trophozoites, which are minor contaminants (<4%) in our purified gametocyte preparations might contribute to the total radioactive DNA measured. For this, identical parasite numbers of asexual stage parasites from gametocyte deficient strain 2.33, and purified gametocytes from wild type strain 2.34 were activated in GAM in the presence of [^3^H]hypoxanthine. After 20 min, label incorporation was observed in gametocytes but not asexual stages (4300 cpm versus 440 cpm). From this we estimate that contaminating asexual stages in our enriched gametocyte preparations only account for approximately 0.4% of the total label incorporation we observed, which is negligible for most applications.

We next tested whether [^3^H]hypoxanthine incorporation into nucleic acids could be improved with the aim to reduce the radioactivity used per assay. As we were using only trace concentrations of [^3^H]hypoxanthine (i. e. 260 nM), we first tested whether addition of non-radioactive (cold) hypoxanthine ranging from 3.1 to 200 μM would increase the overall incorporation of external hypoxanthine (Fig. 1B). As calculated on the basis of the specific activity, total hypoxanthine incorporation indeed increased with the concentration of cold hypoxanthine from 1 to 48 pmol/10^7^ cells (Fig. 1C). However, dilution of label by cold hypoxanthine outweighed this assimilation surplus, resulting in an overall decrease in DNA labelling. Importantly however, the kinetics of label incorporation were identical and independent of the specific activity. The curves showed the same characteristic incorporation pattern described above with an intermediate step at around 6 min and a plateau reached at 12 min post-activation (Fig. 1B). Further experiments were thus performed in media with [^3^H]hypoxanthine as the only source of hypoxanthine. Secondly, we tested whether pre-incubation with radioactive hypoxanthine prior to activation would increase label incorporation. Purified gametocytes were pre-incubated for up to 90 min in the presence of radioactive hypoxanthine before they were activated by transfer to GAM containing the same concentration of [^3^H]hypoxanthine. The pre-incubation did not modify the level of DNA labelling when compared to control cells without pre-incubation (Fig. 1D). At the same time, washing of pre-incubated gametocytes to remove the label, followed by activation in the absence of [^3^H]hypoxanthine did not lead to detectable labelling of DNA (data not shown). These somewhat surprising results indicated that gametocytes did not take up and incorporate [^3^H]hypoxanthine into nucleotides in the resting state (at least during the 90 min before activation). However, upon activation, and perhaps depending upon their emergence from the host erythrocyte, microgametocytes replenished their nucleotide pool at a rate that was high enough to allow rapid detection of [^3^H]hypoxanthine incorporation into DNA from 2 min on, although the precursor has first to be taken up by the parasite and metabolised into nucleotides, before it can be incorporated into DNA. The efficiency of [^3^H]hypoxanthine incorporation only upon gametocyte activation facilitated the assay as cells did not require pre-incubation and could be used directly after purification.

Our experiments revealed interesting insight into the kinetics of DNA replication in the course of gamete formation. Previous studies based on total DNA measurements using Feulgen-stained cells showed a steady increase of DNA over the time of gametogenesis [13]. On the contrary, we observed three distinct stages in most experiments (Fig. 1A and B and data not shown). The first occurrence of radioactive label was generally very weak but could be observed from 2 min after activation. After 4 min, a stronger increase lasted until 5 min when a brief plateau was reached. After another minute, a further surge in radioactive label incorporation lasted until the final plateau was reached after 10 min. We hypothesize that each of the three phases of label incorporation corresponds to an S-phase, when DNA is synthesised, while each intermediate step in the curve likely represents an M-phase, coinciding with a mitotic division. Earlier studies described the formation of mitotic spindles during microgametogenesis with the second mitotic spindle being observed after about 5 min post-activation [15]. This time point corresponds to the brief pause in label incorporation we observed (Fig. 1A), which suggests that the actual M-phase might only take 1 min during which DNA synthesis is stopped. In some experiments, this time point was shifted and occurred between 6 and 7 min but remained clearly distinguishable as a period of reduced DNA synthesis (Fig. 1B). The fact that these distinct stages could be observed in a population of cells indicates that under our experimental conditions, gametocytes were highly synchronised until the point of exflagellation.

**Fig. 2:**
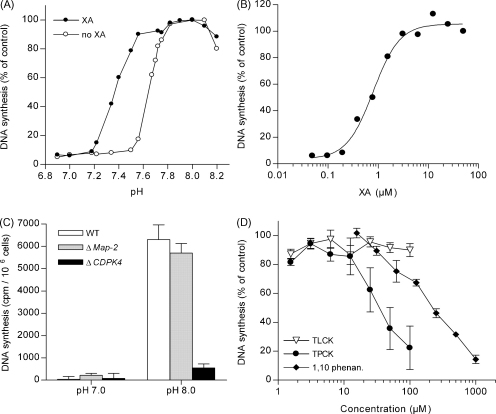
Applications of the [^3^H]hypoxanthine assay. (A) Synergy of XA and pH in gametocyte activation. Purified gametocytes were resuspended in GAM of different pH containing 0.5 μM [^3^H]hypoxanthine either with or without 100 μM XA added. Reactions were stopped after 20 min. The pH of all solutions was adjusted prior to the experiment and confirmed after the experiment. Values are presented as percentage of control at pH 8.0. Shown is one representative experiment of four. (B) Purified gametocytes were activated in GAM at pH 7.4 with varying concentrations of XA. Values are presented as percentage of control at 100 μM XA. Shown is one representative experiment of five. (C) DNA replication of Δ*Map-2* and Δ*CDPK4* parasite strains compared to wild type (WT). Gametocytes were purified, activated in GAM and [^3^H]hypoxanthine incorporation quantified 20 min after activation. Shown is the average and S.E.M. of three independent experiments. The difference in [^3^H]hypoxanthine incorporation in Δ*CDPK4* parasites at pH 7 and pH 8 was not statistically significant. (D) Effect of protease inhibitors on DNA synthesis during gametogenesis. Stock solutions were prepared immediately before the experiment at the following concentrations: 10 mM TLCK (N-a-tosyl-l-lysine chloromethyl ketone) in water pH 3, 10 mM TPCK (N-tosyl-l-phenylalanine chloromethyl ketone) in DMSO, 100 mM 1,10-phenanthroline in DMSO (all from Sigma–Aldrich). Purified gametocytes were activated in the presence of the inhibitors and [^3^H]hypoxanthine incorporation was quantified 20 min after activation. Values are the mean ± SD and are presented as percentage of control without inhibitor (*n* = 3).

Gametocytes can be activated *in vitro* by either a pH shift to the alkaline range or by XA. To date, it is not clear how these two stimuli are recognised by the parasite, but it has previously been shown by counting exflagellation events that addition of XA can extend the permissive pH range for exflagellation, and it was therefore suggested that the two stimuli synergise *in vivo* [16]. To validate our assay, we asked whether the hypoxanthine incorporation would reflect this synergy of pH and XA. Purified gametocytes were resuspended in GAM spanning a pH range of 6.8–8.2 either with or without 100 μM XA added. In the absence of XA, incorporation of radioactive hypoxanthine commenced at pH 7.5 and steadily rose until a maximum was reached at pH 7.8 (Fig. 2A). Addition of XA shifted the curve by 0.3 pH units to the acidic range. Gametocytes were not activated below pH 7.2 even in the presence of XA. We also identified the EC_50_, (effective concentration 50%) of XA, the concentration at which half the cells become activated. Purified gametocytes were resuspended in XA free GAM at pH 7.4 to avoid unintentional activation by alkaline pH alone, and cells were then activated by the addition of different concentrations of XA. The resulting activation pattern followed a concentration dependent curve with an EC_50_ of 1.1 μM (95% confidence interval 0.8–1.4 μM) (Fig. 2B). These results show that hypoxanthine incorporation was intimately linked with the response to activation by pH and XA, thus validating our assay as an appropriate method to quantify gametocyte activation.

After having established the basic parameters of gametocyte activation, we sought to subject parasites with a known exflagellation deficiency to this assay to validate the system as a tool for phenotypic analysis. The two different *P. berghei* parasite lines that seemed suitable were a knock out of the calcium-dependent kinase 4 (Δ*CDPK4*, clone 3.0.7) and a knock out of the Map-2 kinase (Δ*Map-2*, clone 2.4.9), respectively. Neither of the parasite lines exflagellates. Δ*CDPK4* parasites have been described to have a block in the signalling pathway upstream of DNA replication, i.e. gametocytes become activated but fail to initiate DNA replication [10]. Δ*Map-2* parasites have a later block at the stage of cytokinesis [11], i.e. the parasites can replicate their DNA but fail to segregate into gametes. Consistent with this we found that at pH 7.0 neither of the three strains replicated their DNA. When activated at pH 8.0, Δ*Map-2* and wild type parasites showed similar levels of DNA replication, while Δ*CDPK4* parasites did not replicate their DNA (Fig. 2C). Our assay was thus suitable for phenotypic analysis of *P. berghei* mutants.

Our assay is suited for medium-throughput screenings of compounds that interfere with gametocyte activation or DNA replication and thus might have transmission blocking potential. As an example of such an investigation, we chose to analyse the inhibitory effects of three protease inhibitors on DNA replication. The serine/cysteine protease inhibitors TLCK and TPCK as well as the metalloprotease inhibitor 1,10-phenanthroline have been previously shown to block exflagellation centre formation in *P. berghei* [17] and similar effects had been described in *P. falciparum* [18]. However, it is not clear from these studies at which point during gametogenesis these compounds act. We first confirmed that these compounds completely abolished exflagellation under our assay conditions when used at the reported concentrations (100 μM TLCK or TPCK, 1 mM 1,10-phenanthroline) [17] (data not shown). When we applied these compounds in the hypoxanthine assay, we observed potent inhibition of DNA synthesis for 1,10-phenanthroline with an IC_50_ of 200 μM and TPCK with an IC_50_ of 25 μM while TLCK had no effect (Fig. 2D). These results indicate that TPCK and 1,10-phenanthroline inhibit gametogenesis upstream of DNA synthesis, i.e. within the first 2 min after gametocyte activation, while TLCK exerted an inhibitory effect on exflagellation only later, after DNA replication was concluded. The mechanisms through which the different protease inhibitors act on microgametogenesis remain to be identified in more detailed analyses.

In summary, the presented assay that is adapted to the 96-well plate format allows rapid and easy analysis of multiple conditions and provides reproducible information about male gametocyte activation. The assay can be readily used with the same reagents and equipment that are commonly employed for antimalarial growth assays [14] and will thus be useful in drug screenings for transmission blocking studies as well as closer phenotypical analysis of developmental blocks in gametogenesis.
